# Effects of the AMPPS One-on-One Mathematics Intervention on Students’ Complex Computation, Word-Problem Solving, and Math Self-Concept

**DOI:** 10.3390/bs16030432

**Published:** 2026-03-16

**Authors:** Natasha K. Newson, John C. Begeny, Felicia L. Davidson, Robin S. Codding, Kourtney R. Kromminga

**Affiliations:** 1 Department of Psychology, College of Humanities and Social Sciences, North Carolina State University, Raleigh, NC 27695, USA; 2 Department of Applied Psychology, Bouvé College of Health Sciences, Northeastern University, Boston, MA 02115, USA; 3 School of Psychology, College of Education and Human Sciences, University of Southern Mississippi, Hattiesburg, MS 39406, USA

**Keywords:** intervention, dosage, mathematics, computation, math fluency, word-problem solving, math self-concept

## Abstract

Despite consensus in the mathematics education literature regarding the mutually dependent components of math proficiency, as well as the importance of their development, most elementary-aged students in the United States demonstrate a lack of proficiency in math according to national assessment data. Whole number knowledge, which includes skills in computation and word-problem solving, is understood to be a critical foundation for the development of later math skills. This study used a multiple-baseline experimental design to evaluate the impacts of an evidence-based mathematics intervention, Accelerating Mathematics Performance with Practice Strategies (AMPPS), on third- through fifth-grade students’ skills with complex computation, as well as on their word-problem-solving performance. Furthermore, we evaluated effects on students’ math self-concept. Five students identified to have difficulties in math received AMPPS in a one-on-one, in-person format. The results of the study were mixed. For example, when using visual analyses as our primary analytic method, these analyses did not show robust intervention effects on students’ computation skills but did show at least some improvement for most students’ word-problem-solving skills. Additionally, supplemental analyses comparing student growth to national and school-based norms suggested that all participants seemed to benefit from the intervention, but these analyses were not intended to examine experimental causality. Despite study limitations and a lower than optimal number of AMPPS sessions (dosage) provided to students, the present study offers several directions for future research, as well as possible implications for practitioners regarding intervention selection, intensity, and evaluation. The findings will also be discussed in the context of conducting systematic replication studies, which are essential for understanding the generality of a given phenomenon (e.g., an effect of a school-based intervention) across a wide range of situations and conditions.

## 1. Introduction

A strong foundation in mathematics is critical to success later in school (e.g., Duncan et al., 2007; Jordan et al., 2009), as well as later in life (National Mathematics Advisory Panel, 2008; OECD, 2024; Parsons & Bynner, 1997; Vigna et al., 2022). Mathematics literacy is a critical need around the globe, and school-aged students of many countries continue to struggle with math proficiency (OECD, 2024). The United States is one of these countries, and we focus on this country throughout much of this paper, because the setting for our study was in the U.S. To offer just a couple of key statistics on math literacy in the U.S., the most recent national assessment revealed that 61% of fourth graders in the U.S. lack proficiency in mathematics (National Center for Education Statistics, 2024), and this overall pattern of performance in math has remained relatively stable over the past two decades (National Center for Education Statistics, n.d.).

Prior to discussing approaches to improving these low rates of proficiency, one must first understand what it means to be proficient in math. The U.S. National Research Council (2001) proposed five key elements of math proficiency: (1) conceptual understanding, or the knowledge of core math concepts, operations, relations, and properties (e.g., the commutative property, place value within a base-ten system); (2) procedural fluency, or the ability to accurately, efficiently, and flexibly utilize standard algorithms, which inherently relies on automaticity with basic facts; (3) strategic competence, or the ability to formulate, represent (e.g., visually or mentally), and solve problems; (4) adaptive reasoning, or the ability to explain, reflect upon, or justify problem-solving processes (e.g., think alouds; Gersten et al., 2009b); and (5) productive disposition, or the tendency to see math as understandable and valuable, as well as to believe oneself capable of learning math and solving math problems. Importantly, research suggests that these elements are equally important, mutually dependent, and should be focused on simultaneously in math instruction and intervention for students in pre-kindergarten through eighth grade (National Mathematics Advisory Panel, 2008; National Research Council, 2001; Rittle-Johnson & Schneider, 2015; Running et al., 2023). Only with concurrent attention to all five areas can students reliably develop a solid foundation in mathematics that will support them through later schooling and into adulthood (G. Nelson & Powell, 2018; National Mathematics Advisory Panel, 2008; National Research Council, 2001).

Another important consideration when remediating math skill deficits is the treatment intensity, which is commonly examined through variables such as time and group size (Doabler et al., 2019; Miller et al., 2025). Smaller instructor–student ratios increase opportunities for individualized practice and tailored instruction, which have been shown to enhance student responsiveness to interventions (Gersten et al., 2009b). Moreover, within MTSS frameworks, decreasing group sizes is a core mechanism for intensifying support across tiers. Recent meta-analytic findings support this and indicate that group size is a significant predictor of math intervention effectiveness for elementary-aged students (Miller et al., 2025).

### 1.1. Whole Number Knowledge: Importance and Evidence-Based Instructional Strategies

Based on the best available evidence, the National Mathematics Advisory Panel (2008) named whole number knowledge as a critical foundation for the development of later math skills. Whole number knowledge involves the automatic recall of simple operations (i.e., basic facts or addition and subtraction facts with sums and minuends 0–18), fluency with standard algorithms, and a solid sense of number and number relations. Research suggests that automaticity with single-digit problems across all four whole number operations is associated with higher math achievement in subsequent math skills (e.g., Hansen et al., 2017; P. M. Nelson et al., 2016). However, proficiency with complex computation (i.e., multi-digit operations beyond basic facts) requires both automatic recall of basic facts and the application of base-ten knowledge when selecting and executing the appropriate procedures to solve multi-digit problems. Therefore, with complex computation, it is particularly important to consider *how* students solve the problem (i.e., are they using an efficient strategy?). Two common, explicitly taught strategies to solve multi-digit addition and subtraction problems are the traditional standard algorithm, which provides step-by-step procedures for problem solving, and decomposition, which uses place value to decompose the magnitude of numbers before problem solving (Hickendorff et al., 2019). Both strategies require knowledge of the base-ten system, and both are more efficient than direct modeling or counting (National Research Council, 2001).

Related to whole number knowledge, the Common Core State Standards for Mathematics (CCSSM) in the United States indicate that students should be able to add and subtract within 20 and understand place value by the end of second grade and then use their understanding of place value and the properties of operations to solve multi-digit problems by the end of third grade (National Governors Association Center for Best Practices & Council of Chief State School Officers, 2010). However, what is apparent from both the research literature and U.S. national data is that many students are not developing proficiency with whole numbers until much later (National Center for Education Statistics, 2024), and students with poorly developed whole number knowledge are significantly more likely to struggle with later mathematical concepts, such as fractions (Namkung et al., 2018; Resnick et al., 2016). Such evidence suggests that these students are not receiving, or are not benefitting from, timely, targeted intervention.

Also central to whole number knowledge is word-problem solving (National Mathematics Advisory Panel, 2008). The What Works Clearinghouse (WWC), a U.S. government-sponsored source that reviews educational intervention outcomes, recently released a practice guide that identifies six recommendations for effective mathematics intervention that have a strong level of evidence by their standards, one of which is to provide deliberate instruction on word problems (Fuchs et al., 2021). Although key word identification (e.g., difference or all together) is a commonly used strategy, research suggests that utilizing explicit instruction to teach students to identify the underlying structure of a word problem—sometimes referred to as schema instruction—is one of the most effective approaches to word-problem solving (Fuchs et al., 2010; Jitendra et al., 2007; Jitendra & Hoff, 1996; Powell et al., 2020, 2023). Specifically, word problems can be separated into different types or classes based on the action of the problem, not the operation. This is an important distinction, given that early intuitive mathematical problem solving is often completed by directly modeling the action described in a problem (Carpenter et al., 1996). For example, addition and subtraction problems can be broken down into three types: change, combine (or group), and compare (e.g., Fuchs et al., 2014; Jitendra & Hoff, 1996). Within each of these classes, a variety of problems can be developed such that the part of the problem that is unknown varies. Powell et al. (2020) found that students with math difficulties who received schema-based word-problem instruction demonstrated more sophisticated problem-solving strategies and better performance than peers in the comparison group. Additionally, using an attack strategy and diagram, along with explicit instruction, has been shown to improve word-problem-solving performance (Jitendra et al., 2007; Jitendra & Xin, 1997; Powell et al., 2023).

Although computation and word-problem solving are both important pieces of whole number knowledge, research suggests that skills in each domain are not necessarily transferable to one another. Fuchs et al. (2014) found that participation in an intervention focused on computation led to improvements in computation but not word-problem solving and vice versa—that participation in an intervention focused on word-problem solving led to improvements in word-problem solving but not computation. Powell et al. (2015) found some initial evidence of transfer, with students who received supplementary tutoring focused exclusively on word problems demonstrating improvements in both word-problem outcomes and single-digit (but not double-digit) computation; however, like Fuchs et al. (2014), they found that students who received supplementary tutoring focused on computation alone improved in computation but not word-problem outcomes.

Therefore, to fully address whole number knowledge and improve skills among elementary-aged students with low achievement in mathematics, an intervention program seemingly needs to incorporate evidence-based strategies focused on computation, as well as evidence-based strategies focused on word-problem solving. Several researchers have developed interventions that integrate computation and word-problem solving (Jitendra et al., 2007; Powell et al., 2010). Despite the documented need for effective math intervention programs, the research base examining such programs is relatively limited. For instance, a literature review examining the content of all articles published in six major journals on school psychology from 2010 to 2014 revealed that, of the close to 1200 articles published during that timeframe, only 12 (1.0%) focused on math interventions (Villarreal et al., 2017). Moreover, even those programs that have been the focus of research studies may show mixed or no discernable effects. In fact, a recent meta-analysis of math interventions found that, among the 191 unique RCTs examined, the probability that a randomly selected mathematics intervention had an effect of at least 0.25 was 55% (Williams et al., 2022). In other words, there was roughly a 50/50 chance that a selected math intervention had a negligible or otherwise not clearly positive effect.

Additionally, as of early 2025, a search of the WWC’s intervention reports for second- through fifth-grade students yielded only one supplemental mathematics intervention that demonstrated promising effects, and this program is focused exclusively on fractions. Fortunately, the literature base regarding effective instructional strategies focused on whole number knowledge is relatively robust (e.g., Codding et al., 2017; Gersten et al., 2009a, 2009b; National Mathematics Advisory Panel, 2008; National Research Council, 2001), which helps to pave the way for further research and develop effective math intervention programs.

### 1.2. The Accelerating Mathematics Performance with Practice Strategies (AMPPS) Program

One example of an intervention that integrates evidence-based computation and word-problem-solving strategies is the Accelerating Mathematics Performance with Practice Strategies (AMPPS) program, which has been developed as a small-group intervention (AMPPS-SG; Codding & Begeny, 2018b) and an individualized (one-on-one) intervention that can be delivered in person (AMPPS-1:1; Codding & Begeny, 2018a) or virtually. AMPPS was designed to support low-performing students and can be easily integrated within MTSS/RtI models as part of Tier 2 and Tier 3 support.

Initial research evaluating AMPPS is promising. The first published study evaluating AMPPS-SG employed a multiple-baseline design across participants to examine the performance of low-performing second-grade students who received the program as a supplement to their core math curriculum for about 25 min per day, three times per week (Codding et al., 2020). Each group of students moved through three phases: (a) baseline, during which time groups participated in math activities identified by the classroom teacher to align with core instruction; (b) evidence-based instructional strategies from AMPPS-SG; and (c) the full AMPPS-SG intervention (i.e., the same evidence-based instructional strategies as the prior phase, in addition to all AMPPS-SG motivational strategies). All groups received 9–11 weeks of sessions (phases b and c) regardless of the start point in the multiple-baseline design. Immediate effects when moving between phases were not detected; however, this is consistent with prior research suggesting that immediate changes in level are not expected for academic interventions (Barnett et al., 2004). Overall, the results of the study suggested that students benefitted most from receiving the full AMPPS-SG intervention relative to the baseline or instructional strategies only, as illustrated by improvements in mixed addition and subtraction fact fluency according to visual analyses and commonly used effect size criteria.

In a follow-up study, Begeny et al. (2020) examined the relative value of the motivation strategies included in the AMPPS Programs. Second-grade students experiencing difficulties in math received AMPPS-SG as a supplement to their core math curriculum for approximately 25–30 min per day, three times per week. Over the course of 14 weeks, student groups moved through the following phases of a multiple-baseline design: (a) instructional components only; (b) instructional components in addition to goal setting, performance feedback, and reinforcement for performance; and (c) the full AMPPS-SG intervention (i.e., all components of phase b, in addition to a group-based reward contingency). Consistent with the results of Codding et al. (2020), student groups in this study tended to not show immediate effects when moving between phases but showed a similar overall pattern of improved performance on mixed addition and subtraction probes during the phase in which they received the full AMPPS-SG intervention relative to prior phases. Meaningful improvements were observed with visual analyses and statistically significant effect size criteria.

A third study evaluated the effects of AMPPS-1:1 when delivered virtually using a multiple-baseline design across participants (Newson et al., 2025). Second- and third-grade students with math difficulties moved through two phases: (a) baseline, during which time they received an evidence-based reading intervention delivered virtually, and (b) intervention, during which time they received AMPPS-1:1 via Zoom. Students received approximately 20 min sessions three times per week over the course of 10 weeks during the summer months. As expected, immediate effects were not detected upon moving to the intervention phase (Codding et al., 2020; Ledford et al., 2018). However, visual analyses and commonly used effect size criteria suggested significant improvements in mixed addition and subtraction fact fluency for all but one student.

Of note, the existing AMPPS literature has focused specifically on fluency with basic addition and subtraction facts (i.e., numerals ranging from 0 to 18). However, no published research to date has specifically examined the impact of AMPPS on (a) students’ fluency with complex addition and subtraction problems or (b) students’ word-problem-solving performance. Evaluating these outcome variables would be a valuable contribution to the growing evidence base surrounding AMPPS because the program contains units specifically focused on word-problem solving, as well as complex addition and subtraction. Therefore, an important next step in mathematics intervention work, and AMPPS research specifically, would be to examine both complex computation and word-problem solving as separate outcome variables.

### 1.3. Productive Disposition, Self-Beliefs, and Performance

As stated above, productive disposition was highlighted as an essential element of mathematics proficiency by the National Research Council (2001). Despite additional calls for researchers to examine motivation and affect in math education and intervention (e.g., Begeny et al., 2020; Codding et al., 2017; Gersten et al., 2009a), productive disposition is often not included in empirical evaluations of mathematical proficiency, perhaps as a result of disagreements on how to best operationalize and subsequently measure the construct (Philipp & Siegfried, 2015).

Although productive disposition, specifically, has not received significant focus in the empirical literature, one’s attitude toward or self-beliefs regarding mathematics have received substantial attention, and research repeatedly highlights their relationship with math performance (e.g., Ma & Kishor, 1997; Valentine et al., 2004). More recent longitudinal research suggests that the relationship is not just correlational in nature but reciprocal and predictive, such that math performance predicts later math self-beliefs and these math self-beliefs, in turn, predict subsequent math performance (Arens et al., 2017; Ganley & Lubienski, 2016; Wu et al., 2021).

Increased conceptual understanding is thought to lead to gains in confidence (National Research Council, 2001); therefore, it stands to reason that intervention programs that target conceptual understanding may contribute to a more positive attitude toward mathematics and improved self-beliefs. In fact, there is emerging evidence that intervention programs can improve students’ self-beliefs regarding their math skills. A recent review of math tutoring programs found that seven out of the eight studies reviewed observed increases in students’ math confidence (Place et al., 2023). This review also highlighted a relationship between math confidence and performance, such that the programs that demonstrated the largest increases in confidence also demonstrated the largest increases in performance. Although not a causal relationship, this finding further bolsters the notion that self-beliefs and performance are, indeed, related and that self-beliefs may be worth considering as an outcome variable in mathematics intervention research to better understand the full scope of a program’s effects.

It is worth noting that research surrounding attitudes and self-beliefs is somewhat muddied by inconsistently defined and utilized terminology, such as confidence, self-concept, self-esteem, and self-efficacy. Despite the considerable overlap between these constructs, some have highlighted theoretical distinctions between the two most popular terms—self-concept and self-efficacy (Bong & Skaalvik, 2003; Zimmerman, 2000)—and more recent research has focused on empirical distinctions (Lee, 2009; Marsh et al., 2019). Math self-efficacy is both context-specific and situationally specific and is often measured by presenting a problem and then asking the respondent how confident they are that they can solve the problem correctly (Bong & Skaalvik, 2003). Math self-concept, on the other hand, is broader but still context-specific, reflecting an individual’s perception of their overall math abilities. As self-concept seems to be more closely aligned with a component of the National Research Council’s (2001) definition of productive disposition (i.e., “to see oneself as an effective learner and doer of mathematics”, p. 131, National Research Council, 2001), “self-concept” is used in this study to describe students’ beliefs about their own math abilities.

### 1.4. Purpose of the Present Study

Given the importance of improving elementary-aged students’ math proficiency and addressing students’ math self-concept, this evaluation of AMPPS-1:1 aimed to answer the following research questions.

RQ.1 Do students who receive AMPPS-1:1 show improved fluency with complex addition and subtraction?

RQ.2 Do students who receive AMPPS-1:1 demonstrate improvements in their skills with word-problem solving?

RQ.3 Are there indicators of improved math performance across the duration of the intervention as measured by pre–post analyses? These exploratory analyses aligned with RQ3 are valuable to our overall study, but the analyses did not specifically examine causality due to our selected research design and sample size.

RQ.4 Do students who receive AMPPS-1:1 report improved math self-concept at the end of the intervention period?

## 2. Materials and Methods

### 2.1. Participants and Setting

Participants were recruited from an urban elementary school located within a large school district in the Southeast United States. The student population in the school was 52% male and 48% female, with racial demographics reported as 45% White, 26% Latine, 23% Black, 4% two or more races, and 2% Asian or Pacific Islander. In addition, 39% of the students were eligible to receive free and reduced-price lunches.

Third- through fifth-grade students who were identified to have difficulties in math, as defined by a multiple-method gated screening process (described later), were invited to participate in the study. In collaboration with school personnel, seven students were invited to participate. Parental consent and child assent were obtained for all seven participants. Due to frequent absences, resulting in 10 or fewer total AMPPS sessions, two students did not receive the intervention as intended. As a result, the final sample included five students. Three participants identified as White, one as Black or African American, and one indicated that they preferred not to respond. Three participants (Students 1, 3, and 4) were in the third grade, one was in the fourth grade (Student 2), and one was in the fifth grade (Student 5). One student (Student 2) received reading services under an IEP at the time of the study. None of the students concurrently received intensive support in mathematics. English was the primary language spoken at home for all five students, and none of the students received English language services. All intervention sessions were conducted in a room or hallway within the participating school, which were the typical locations for supplemental intervention.

All participants received math instruction as part of their core curriculum in school. The school utilized a district-developed math curriculum that, for each new instructional topic, included a launch, review, mini-lesson with discussion, and practice. Teachers utilized slide decks provided by the school district to aid in direct instruction. Physical manipulatives were commonly utilized across grades and topics.

### 2.2. Materials

#### 2.2.1. Assessment Measures

**Acadience Level 2 Computation Progress Monitoring Probes (A-PM).** The Acadience Level 2 Computation Progress Monitoring Probes (Wheeler et al., 2019) are 2 min timed multi-skill tests in which students are asked to answer as many problems as they can. There are 20 A-PM probes available, each containing 20 problems, including 1 × 1, 2 × 1, 2 × 2, and 2 × 2 × 2 × 2 addition and subtraction problems with and without regrouping, all of which are written vertically. Of note, and as will be discussed in more detail later, no participants received the unit of AMPPS targeting 2 × 2 and 3 × 3 computation with regrouping; therefore, at least 35% of the problems on the A-PM probe were mismatched to the instructional content received, depending on how far students progressed in the curriculum. The probes are not grade-specific. The measure is scored by counting each correct digit, rather than each correct answer. Each correct digit is given one point, and points are summed for a total score. The total score, when divided by two, provides the digits correct per minute (DCPM). Test–retest reliability is 0.77 and alternate-form reliability is 0.78 (Gray et al., 2019b). Compared to the SAT10 Total Math Score, predictive validity is 0.72 and concurrent validity is 0.68 (Gray et al., 2019b).

**Acadience Grade-Level Computation Benchmark Assessment (A-Comp).** Like the A-PM, the Acadience Grade-Level Computation Benchmark Assessment (Wheeler et al., 2019) is a timed test in which students are asked to answer as many problems as they can. Each A-Comp probe has 20–25 problems, and the content varies by grade level. The third-, fourth-, and fifth-grade probes include addition, subtraction, multiplication, and division problems, and students are given 3, 5, and 6 min, respectively, to complete the assessment. Addition, subtraction, and multiplication problems containing whole numbers are written vertically, while addition and subtraction problems containing fractions are written horizontally. Division problems are written with a division bracket. The measure is scored by counting each correct digit as one point and totaling the number of points earned. Aligned with guidance from Acadience (Wheeler et al., 2019), two probes were administered at each time point, and the points earned on each probe were averaged to generate a final score. Across third- through fifth-grade probes, test–retest reliability coefficients range from 0.81 to 0.90 and alternate-form reliability coefficients range from 0.73 to 0.88 (Gray et al., 2019b). Compared to the SAT10 Total Math Score, predictive validity coefficients range from 0.71 to 0.73 and concurrent validity coefficients range from 0.69 to 0.76 (Gray et al., 2019b).

**Curriculum-Based Measure of Word Problem Solving (CBM-WPS).** The Curriculum-Based Measure of Word Problem Solving is a measure designed to evaluate students’ word-problem-solving skills (Jitendra et al., 2005, 2014; Leh et al., 2007). The original set of CBM-WPS probes developed by Jitendra and colleagues includes six probes, each of which contains eight addition and subtraction word problems (6 one-step problems and 2 two-step problems). Each problem meets the semantic criteria for one of the three types of addition and subtraction problems (i.e., change, combine/group, compare), and the position of the unknown quantity varies across problems. One- and two-digit numbers are utilized in the problems, and no problems include distractors. Students are allotted 10 min to complete the measure. The CBM-WPS measure is scored by awarding one point for each correct number sentence and one point for each correct answer, including the correct label. As such, one-step problems can earn a maximum of two points (number sentence, answer with label), and two-step problems can earn a maximum of four points (number sentence for the first step, answer for the first step, number sentence for the second step, and final answer with label). The number of points earned across all problems is then totaled. Internal consistency estimates range from 0.60 to 0.83, and concurrent and predictive validity coefficients are statistically significant, ranging from moderate to weak across a variety of different measures (e.g., the Problem-Solving subtest of the SAT-9, MAP mathematics; Jitendra et al., 2005, 2014; Leh et al., 2007).

To have enough probes for progress monitoring throughout the baseline and intervention phases of the study design, we created additional probes based on those developed and evaluated by Jitendra and colleagues. Additionally, to accommodate time restraints resulting from the participating school’s designated intervention time blocks (30 min), the probes were shortened to include 4 one-step problems and 1 two-step problem, with an administration time of 5 min per probe. To preserve content validity, questions were moved and removed strategically so that each set of three consecutive probes covered all possible combinations of problem types and operations, as opposed to this being accomplished every two probes with the original measure.

**AMPPS Placement Assessment.** The AMPPS Placement Assessment was developed to assist with determining the appropriate starting point for students in the AMPPS Curriculum. The Placement Assessment has eight levels, one associated with each unit of the AMPPS Curriculum. Each level includes a 1 min timed computation probe and three word problems aligned to the content of the associated unit. Based on a student’s performance on Level A of the Placement Assessment, the decision is made to either (a) have the student begin with Unit A of the AMPPS Curriculum or (b) administer Level B of the Placement Assessment. This decision-making process is repeated until an appropriate start point is determined. Psychometric data are not yet available for the AMPPS Placement Assessment; however, implementation experience with AMPPS suggests that the measure is a useful secondary tool for ensuring that students start at an appropriate place within the AMPPS curriculum.

**Self Description Questionnaire-I (SDQI).** The Self Description Questionnaire-I (Marsh, 1990) is commonly used to measure self-concept among elementary-aged students (e.g., Arens et al., 2011; Byrne, 1996; Ganley & Lubienski, 2016; Han, 2019). Within the domain of math self-concept, the SDQI contains items related to self-perceptions of competence, as well as items related to interests and affect. Only items related to competence were utilized in the present study because research has emerged since the original development of this measure suggesting that competency and affect are distinct constructs (Marsh et al., 1999) and that competency items are more related to achievement than affect items (Arens et al., 2011). Furthermore, following the precedent set in previous research (Han, 2019), the negatively worded item related to competence (i.e., *I am dumb at math*) was not included given research suggesting that younger children may experience difficulty responding to negatively worded items (Marsh, 1986), as well as factor analyses showing that models eliminating negative items on the SDQI demonstrated a better fit than those including negative items (Arens et al., 2011). The remaining items related to self-perceptions of math competency that were utilized in the present study are as follows: *I get good grades in math*, *I learn things quickly in math*, *I am good at math*, and *Work in math is easy for me*. Each item asks students to respond on a five-point Likert scale (*true, mostly true, sometimes false and sometimes true, mostly false, false*). Internal consistency for the total math scale, including positively and negatively worded items related to competency and affect, is 0.89 (Marsh, 1990). For the math competency items alone, one prior research study suggests that the internal consistency ranges from 0.76 to 0.87 for third- through fifth-grade students when both positively and negatively worded items are included (Marsh et al., 1999), while another found internal consistency to be 0.92 for third- through sixth-grade students when only positively worded items are included (Arens et al., 2011).

#### 2.2.2. Intervention Materials

The version of AMPPS that was used in this study was specifically designed for one-on-one (student–instructor) instruction in an in-person context, and it utilized the second edition of the AMPPS-1:1 curriculum (Codding et al., 2022). Based on the results of the AMPPS Placement Assessment, all five participants began in the final unit focused on simple computation (i.e., addition and subtraction 11 to 18). Following the completion of this unit, students progressed to subsequent units focused on complex computation (i.e., 1 × 1 × 1 addition, 2 × 1 addition and subtraction; some students received at least some lessons in the unit covering 2 × 2 and 3 × 3 addition and subtraction without regrouping). Connected to the research questions in this study and selected progress monitoring evaluation tools for computation, it was hoped that students would then progress to the unit focusing on 2 × 2 and 3 × 3 addition and subtraction with regrouping, but none of the students reached this unit during the study. Appendix A shows each unit of AMPPS.

Materials for each unit include (a) an implementation protocol, which includes an implementation flow chart and a materials list, as well as a brief activity description and key comments for each procedural step; and (b) instructional materials such as flashcards, number lines, standard algorithm worksheets, decomposition worksheets, and word-problem diagrams. A Google Sheet containing a graph and tracking form to monitor student progress and a Star Chart (Begeny et al., 2010) for motivational procedures were also maintained for each student.

### 2.3. Procedures

#### 2.3.1. Screening and Pre–Post Assessments

All students in third, fourth, and fifth grade at the participating school (*N* = 141) were screened with the middle-of-year (i.e., Winter) A-Comp, which is a normed grade-level assessment. Those who scored at or below the 45th percentile and were identified by school personnel as a potentially good fit for the program (*n* = 37) were assessed with the AMPPS Placement Assessment to further evaluate each student’s fit and starting point with the AMPPS program. None of the students assessed were placed within the units targeting complex addition and subtraction; therefore, seven students who were placed within the unit immediately preceding those targeting complex addition and subtraction (i.e., the unit focused on addition and subtraction 11 to 18) were selected to participate. Of note, all five students in the final sample performed in the well-below-benchmark range on the middle-of-year A-Comp.

Additionally, to ensure that they would be able to read the word problems utilized in the intervention and progress monitoring probes, students’ performance on middle-of-year reading benchmark assessments was examined. All students’ oral reading fluency (ORF) scores fell in the below- or at-benchmark ranges. Word problems included in the AMPPS Curriculum are written at or below a level associated with the beginning of second grade, and word problems included in the progress monitoring probes are written at or below a third-grade level. Additionally, students were given the option to have word problems read aloud to them, both during the intervention and while completing the progress monitoring probes. Therefore, there were no concerns regarding students’ ability to read the presented word problems.

Students completed the end-of-year (i.e., Spring) A-Comp during the school’s benchmark testing period, which was within 2.5 weeks of their last AMPPS intervention session (i.e., post-intervention). Finally, the SDQI was administered to all participants prior to baseline and post-intervention.

#### 2.3.2. Training Interventionists

Interventionists were undergraduate students in psychology, all of whom were previously certified in and had at least one semester of experience implementing the Helping Early Literacy with Practice Strategies (HELPS) program, an evidence-based reading intervention (Begeny et al., 2010, 2011). The lead author of this study is a Certified AMPPS Trainer and was responsible for training the interventionists to implement the AMPPS-1:1 intervention procedures. Interventionists practiced implementation by taking turns posing as the interventionist or student. Implementation mastery was determined only when each interventionist could implement the full protocol independently and with 100% integrity on both the core implementation procedures and the tips and reminders checklist (described later) during a mock session. All interventionists were required to meet these criteria before being able to implement the protocol with a student participant, and implementation integrity with student participants was continuously monitored (described later).

#### 2.3.3. AMPPS Intervention

One sub-lesson is implemented per instructional session, which lasts approximately 20 to 25 min. AMPPS instructional components and strategies include modeling, corrective feedback, guided practice, visual representations, explicit timing, flashcard drills, think alouds, cumulative review, and progress monitoring—all of which have evidence for improving students’ math skills (for additional detail, see Newson et al., 2025). The majority of intervention components are consistent across each AMPPS session, and they include the following: (a) the student engages in an activity to promote conceptual understanding (e.g., commutative property, “thinking addition” to solve subtraction, skip counting); (b) the student practices solving problems using an instructional strategy (e.g., counting on or counting back with a number line, making ten with ten frames, standard algorithm, decomposition); (c) the interventionist facilitates guided practice and monitors independent practice with word problems; (d) the student responds orally to a flashcard drill or engages in a think aloud; (e) the student completes a 1 min timed curriculum-embedded assessment focused on computation (i.e., a “Sprint”); (f) the interventionist leads the student in correcting the Sprint; (g) the interventionist graphs the student’s problems correct per minute (PCPM) and problems incorrect per minute (PIPM) and provides praise and feedback; and (h) the interventionist awards the student stars on a Star Chart based on effort and performance (i.e., meeting their “Sprint Challenge”, meaning that their PCPM was higher than during the previous session). The sub-lessons within the Review Lesson (Lesson 3 of all units) replace steps a to d listed above with a review game and replace step f with a curriculum-embedded assessment focused on word problems. Some examples of materials utilized during intervention delivery include flashcards, number lines, ten frames, and worksheets designed to support computation using the standard algorithm, computation using decomposition, and word problem solving. All sub-lessons are completed sequentially until the Review Lesson, at which point student performance is utilized to determine whether the student should progress to the subsequent unit or receive additional practice within the current unit. Mastery is assessed with the curriculum-embedded assessments, which are aligned to the skills of the lesson. If a student meets the unit-specific problems correct per minute, problems incorrect per minute, and word-problem accuracy criteria during a sub-lesson within the Review Lesson, the student advances to the next unit in the curriculum. If a student does not meet the unit-specific criteria, an additional sub-lesson within the Review Lesson is completed, and up to three review sub-lessons can be completed per unit. If a student completes all three review sub-lessons, they proceed to the next unit, regardless of whether they meet the unit-specific criteria.

#### 2.3.4. Progress Monitoring of Students’ Fluency with Complex Addition and Subtraction

A-PM probes were used for progress monitoring to evaluate progress in fluency with complex addition and subtraction throughout the intervention. During both the baseline and intervention phases, A-PM probes were administered every 4–5 days. The data collected from these progress monitoring probes served as the dependent variable of interest related to RQ1 within the multiple-baseline experimental design.

#### 2.3.5. Progress Monitoring of Students’ Word-Problem-Solving Performance

CBM-WPS probes were used for progress monitoring to evaluate progress in word-problem-solving performance throughout the intervention. Prior studies utilizing this measure administered probes once every 2 weeks, with the rationale that instruction focused on word-problem solving occurs less frequently than instruction focused on computation (Jitendra et al., 2005, 2014; Leh et al., 2007). However, given that word-problem instruction and practice occur during every AMPPS session, CBM-WPS probes were administered every 4–5 days across the baseline and intervention phases. The data collected from these progress monitoring probes served as the dependent variable of interest related to RQ2 within the multiple-baseline experimental design.

### 2.4. Experimental Design and Conditions

This study employed a single-case experimental design (SCD) that aligned with the highest standards and recommendations for a methodologically rigorous SCD, as described by the U.S. Department of Education’s Institute for Education Sciences (IES): (a) the outcome measure had face validity; (b) interscorer agreement was collected for at least 20% of the data points for each outcome measure; (c) the outcome measure was not overly aligned with the intervention; (d) consistent data collection procedures were utilized across phases; (e) the outcome measure was independent of the intervention; (f) no known confounding factors were present; and (g) the independent variable was systematically manipulated (What Works Clearinghouse, 2022).

A multiple-baseline across-participants design was the type of SCD used to answer RQ1 and RQ2 and assess for causality. The multiple-baseline design likewise aligned with all but one of the highest standards and recommendations for methodological rigor recently offered by the IES (What Works Clearinghouse, 2022). Specifically, it (a) included a minimum of three cases (i.e., participants); (b) included at least five data points in the intervention phase; (c) ensured that all data were vertically comparable; (d) ensured that all participants had baseline data collected prior to the introduction of the intervention to any participant; and (e) ensured that participants still in baseline remained in baseline at or after the time point at which the preceding participant began the intervention. The only recommendation that could not be fully implemented due to some logistic constraints was the inclusion of at least six data points in the baseline for at least three participants. However, one of our participants had six baseline data points and all participants had three or more (a long-established criterion for sound SCDs), and, collectively, the rigor of our design was relatively strong compared to the many published SCDs in the behavioral sciences.

The study design included a baseline condition where students did not receive AMPPS-1:1 but received a brief (i.e., 5–7 min) subitizing activity. The baseline condition was followed by the intervention condition, where students received AMPPS 2–3 times per week for 7–9 weeks, resulting in a range of 15–21 total AMPPS sessions for each student.

AMPPS participants were randomly assigned to the design’s staggered intervention start points (i.e., 3, 4, 5, or 6 baseline data points). This procedure for randomization within a multiple-baseline across-participants design is consistent with recommendations and other recent studies (e.g., Begeny et al., 2023b; Bice-Urbach & Kratochwill, 2016), and it has been utilized to strengthen the validity of single-case designs (Kratochwill & Levin, 2010; Levin & Ferron, 2021). To account for potential baseline trends, a plan was developed to keep students who exhibited an increasing trend in their A-PM baseline data in the baseline condition until their data stabilized or until 10 baseline data points had been collected, whichever came first. The potential need to begin the intervention condition after no more than 10 baseline data points was due to a desire to provide targeted math intervention as soon as possible to students who need it. However, this modification to randomization was not required, as no students demonstrated notable increasing trends in their A-PM baseline data. Because there were two measures being used in this multiple-baseline design, decisions about deviations from randomization were based only on one of the measures for consistency and efficiency. The A-PM was chosen as the measure upon which to base such decisions given that more is known about the psychometrics of computation measures compared to word-problem-solving measures.

### 2.5. Interscorer Agreement

A member of the research team independently rescored 100% of the A-PM and CBM-WPS probes, and interscorer agreement was calculated by dividing the number of agreements on points by the number of agreements plus disagreements and multiplying by 100. Mean agreement on the A-PM across the baseline and intervention phases was 99.1% (*SD* = 0.03, range = 81.8–100%). Mean agreement on the CBM-WPS across both phases was 98.1% (*SD* = 0.04, range = 83.3–100%).

### 2.6. Intervention Fidelity

A member of the research team who was trained in all AMPPS-1:1 procedures observed 30% of the implemented sessions to assess interventionists’ fidelity in implementing AMPPS. Intervention fidelity was assessed by using checklists to evaluate both intervention adherence (i.e., whether the core procedures were implemented as intended) and intervention quality (i.e., how well the procedures were implemented; Sanetti & Collier-Meek, 2014). As shown in Appendix B, the example checklist for AMPPS (Unit G) specifies core procedures at the top and items related to intervention quality immediately afterward. Across interventionists, the mean intervention adherence was 96.5% (*SD* = 0.06, range = 80.0–100%) and the mean intervention quality was 93.3% (*SD* = 0.05, range = 83.3–100%).

It is also important to consider intervention fidelity in terms of dosage (i.e., the frequency, duration, and cadence by which the intervention is implemented as recommended by the intervention developers). In this study, students received AMPPS according to the prescribed session duration (i.e., a complete session) when a session was implemented, but, as we will describe later in the Discussion, students did not end up receiving the currently recommended number of AMPPS sessions throughout the course of the study (i.e., it is recommended that students receive 30–50 total sessions, but, as noted above, students received 15–21 total AMPPS sessions). Students therefore received a high enough dosage to justify an analysis of the effects of AMPPS on students’ performance given the dosage received, but we highlight here that the dosage was lower than recommended and in comparison to past AMPPS studies.

Related to the total number of AMPPS sessions that participants received, the cadence of implementation was sometimes also lower. It is generally recommended that students receive AMPPS at least three times per week (which is how students were scheduled in this study), but, due to factors such as student absences, students often only received AMPPS twice per week on average. Overall, the intervention fidelity was sound and allowed for a methodologically rigorous evaluation of AMPPS, but dosage factors meant that it was a methodologically rigorous examination of AMPPS when students received ~15–21 total sessions—which is less than what the AMPPS developers currently recommend.

### 2.7. Data Analysis Strategy

To address RQ1 and RQ2, respectively, performance on the A-PM and CBM-WPS probes across the baseline and intervention phases was graphed for each student and analyzed visually. Consistent with recommendations and standards for SCD data analysis (e.g., Kratochwill et al., 2013), the level, trend, variability, immediacy of effect, overlap, and consistency of data patterns within and between phases were examined. Additionally, nonoverlap of all pairs (NAP) effect sizes were computed to supplement the visual analysis. NAP was selected because, by the nature of considering all possible comparisons of baseline and intervention data points, the calculation reduces the effects of small amounts of data and outliers (Parker & Vannest, 2009). NAP was calculated using Vannest et al. (2016) web-based application. Interpretations of NAP effect sizes are as follows: small (0–0.65), medium (0.66–0.93), and large (0.94–1.00; Parker & Vannest, 2009).

Additionally, in accordance with RQ3, overall growth in students’ math skills across the duration of the project was examined. The change over time on the A-Comp was reported and interpreted according to both school-based and national norms, including comparisons with school-based norms of students with low math performance who did not receive AMPPS. Of note, given our SCD and the small sample of students who received AMPPS, RQ3 was exploratory and descriptive in nature, and, unlike the visual analyses for RQ1 and RQ2, analyses comparing participants’ performance to school and national norms did not examine experimental causality. Finally, descriptive statistics were utilized for students’ reported math self-concept pre-baseline and post-intervention to examine RQ4. These results were interpreted according to the Likert scales associated with the respective survey items.

## 3. Results

The findings are discussed according to each research question and, within these questions, according to the different forms of analysis.

### 3.1. Do Students Who Receive AMPPS-1:1 Show Improved Fluency with Complex Addition and Subtraction?

#### 3.1.1. Visual Analyses

**Figure 1:**
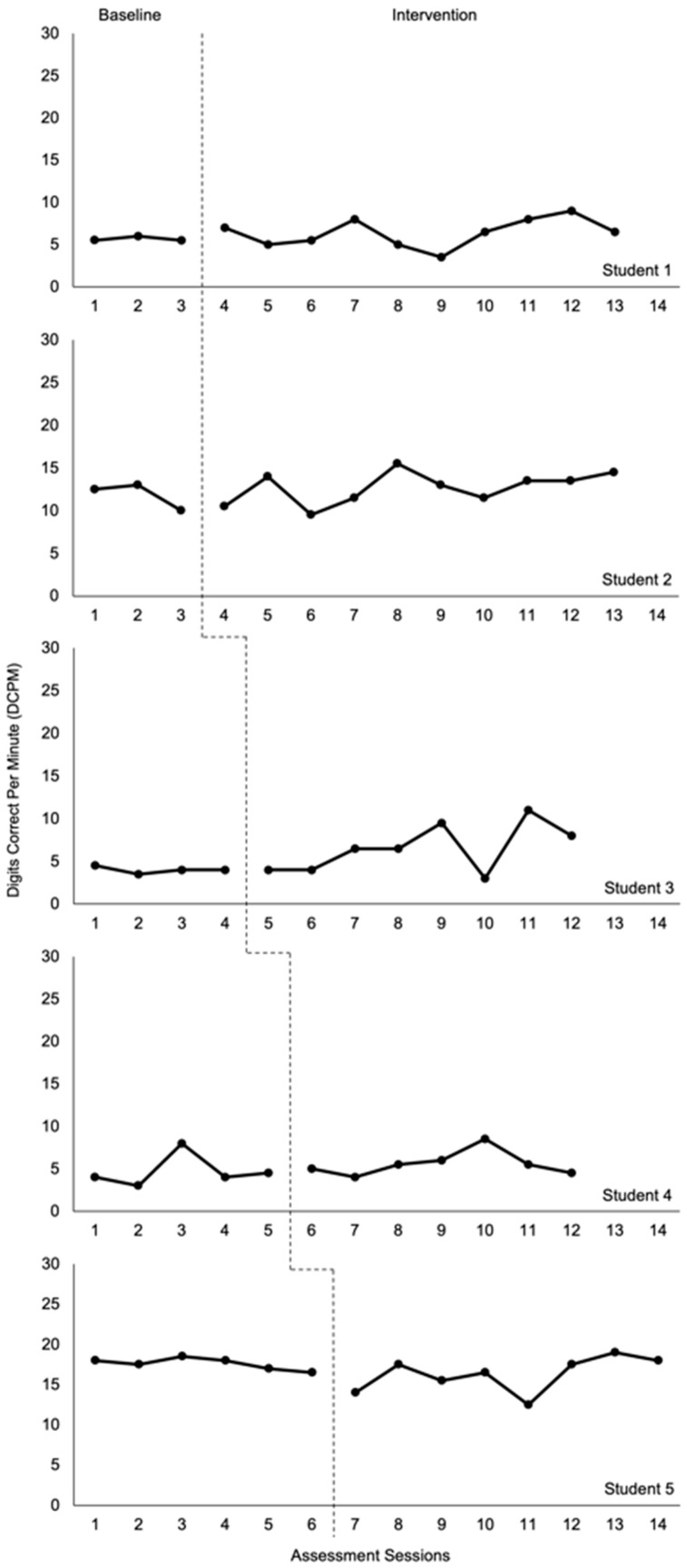
Digits correct per minute (DCPM) on A-PM across phases.

Figure 1 shows the digits correct per minute (DCPM) for each student across the baseline and intervention conditions. Although it can depend on the specific types of skills being learned, immediate changes in the level of performance at the onset of an academic intervention are not expected because the student is learning new skills; instead, improvements in trend and variability are gradual over time (Barnett et al., 2004; Ledford et al., 2018). Consistent with this expectation, students in our study did not have any immediate changes in level from baseline to intervention; however, data variability and trend changes were noted.

Students 1, 3, and 5 each had flat baseline data and then more variable intervention data, with Students 1 and 3 having slightly higher levels of performance during the intervention compared to the baseline and Student 3 showing an overall increasing trend (except for one data point that notably dropped during assessment session 10). Student 5 had a slightly lower level of performance during the intervention relative to the baseline, but an increasing trend emerged during the last four assessment sessions.

For Student 2, the baseline and intervention data had some degree of variability, but the intervention also showed a slight increasing trend in performance over time and a minor increase in level. Student 4 likewise had some variability in performance during the baseline and intervention, with only a slight increase in level during the intervention. Table 1 includes the A-PM mean scores for each student and condition, which likewise shows a small increase in level (based on mean performance) for Students 1–4 and a slight decrease for Student 5. The changes were quite small, however, with Student 3 being the only student who had a change in mean performance of more than 1 DCPM. Overall, visual analyses of the computation data suggest that students’ performance on the A-PM did not meaningfully change from baseline to intervention, and a functional relationship was not shown.

#### 3.1.2. NAP

Table 2 provides effect size comparisons between computation performance in the baseline and intervention phases for each student. NAP analyses indicate no statistically significant effects for any of the individual students. These results are consistent with the visual analyses. However, in the NAP analysis that considered all students’ performance collectively (bottom row), there was a small but statistically significant increase in students’ overall performance during intervention. This finding will be discussed later.

### 3.2. Do Students Who Receive AMPPS-1:1 Demonstrate Improvements in Their Skills with Word-Problem Solving?

#### 3.2.1. Visual Analyses

**Figure 2:**
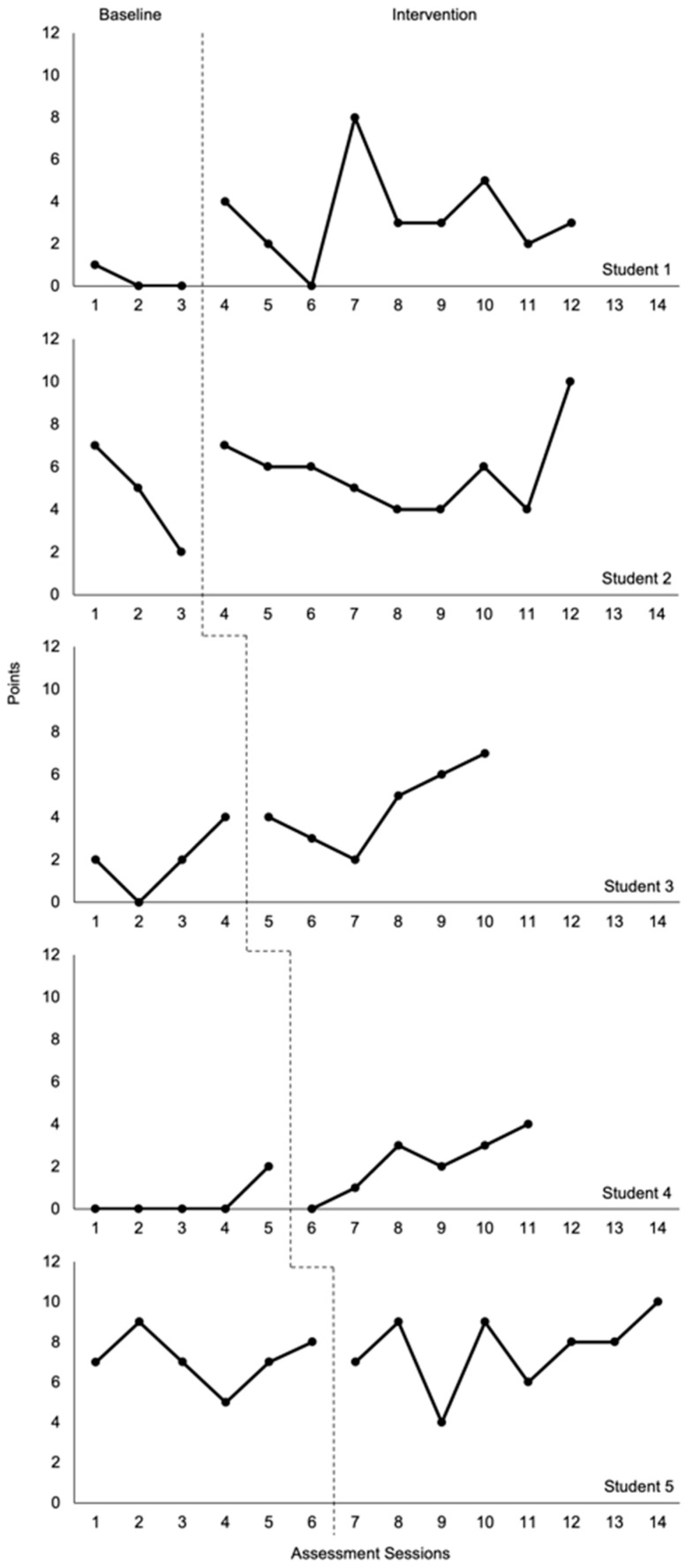
Points on CBM-WPS across phases.

Figure 2 shows the points earned on the CBM-WPS for each student across the baseline and intervention conditions. Baseline performance was relatively stable for Students 1 and 4, while Student 2 showed a decreasing trend, Student 3 an increasing trend, and Student 5 substantial variability.

During the intervention, Students 1, 2, 3, and 4 showed overall improvements in performance on the CBM-WPS measure. Student 1 had a rather immediate increase at the start of the intervention, and, although the data were quite variable throughout the intervention, the overall level of performance was notably higher than during the baseline. The intervention data for Students 2, 3, 4, and 5 generally started at levels that were consistent with their respective baseline data, but each student eventually showed an increasing trend. However, the increasing trend for Student 5 followed a lot of variability and was still consistent with their baseline performance. In this way, the visual analysis showed that Student 5 did not seem to improve on the CBM-WPS measure during the intervention.

Table 1 provides a comparison of the means across conditions for each student. Consistent with our visual analysis describing improved levels and/or trends for Students 1, 2, 3, and 4, the mean intervention data for these students likewise reflect improvements during the intervention. With Student 5, the improvement during the intervention was much smaller (only 0.4 points), although it should also be noted that Student 5 clearly had the strongest baseline performance across all students and perhaps notable growth during the intervention for this student would have required more time and intervention sessions.

#### 3.2.2. NAP

Table 2 provides effect size estimates of word-problem-solving performance in the baseline phase compared to the intervention phase for each student. NAP analyses indicate a medium effect size that was statistically significant in the expected direction for Student 1. Improvements from baseline to intervention for Students 3 and 4 yielded a medium effect size that approached statistical significance at *p* < 0.05. These results are generally consistent with the visual analyses, particularly the noted relatively larger improvements in level from baseline to intervention for Students 1, 3, and 4. Finally, across all students, a medium statistically significant effect was detected on the CBM-WPS measure.

### 3.3. Are There Indicators of Improved Math Performance Across the Duration of the Intervention as Measured by Pre–Post Analyses?

Table 3 shows exploratory analyses using school-based norms for performance on the A-Comp. These data were collected at the participants’ school during the same year that our study took place. As such, the data reflect the benchmark assessment scores of participants’ peers by grade level. Table 4 shows participants’ performance on the A-Comp, as well as nationally normed data for expected performance at EOY.

Compared to school-based norms, four out of the five student participants (everyone except Student 4) improved more on the A-Comp from MOY (administered prior to the baseline condition; pre-test) to EOY (administered at the end of the intervention condition; post-test) than their grade-level peers. We also analyzed participants’ performance in comparison only to non-participant students from the same school who likewise demonstrated math difficulties during the MOY assessment (i.e., scored well below benchmark at MOY). From these analyses, the same four participants exceeded expected growth relative to their grade-level peers who likewise evidenced difficulty in math at MOY. Finally, compared to national norms, all five study participants showed greater improvements than their grade-level peers.

### 3.4. Do Students Who Receive AMPPS-1:1 Report Improved Math Self-Concept at the End of the Intervention Period?

Table 5 summarizes students’ responses for each item of the SDQI. Overall, changes from pre- to post-test were minimal, suggesting that students did not report higher math self-concept at the end of the study.

## 4. Discussion

The purpose of this study was to evaluate the impact of receiving an evidence-based math intervention (AMPPS-1:1) on students’ computation fluency and on their word-problem-solving skills. Both computation and word-problem solving are essential components of whole number knowledge, a widely agreed upon foundation for later math skills. Yet few studies consider both computation and word-problem solving as outcome variables in tandem. Overall, the findings of the present study were mixed regarding the impacts of AMPPS-1:1 on students’ computation and word-problem solving.

According to visual analyses, which was the primary analytic approach in the present study, students’ computation performance did not significantly change in response to receiving the intervention. This finding is inconsistent with several prior studies that demonstrate improvements in computation skills following AMPPS-1:1 (Newson et al., 2025) or AMPPS-SG (Begeny et al., 2020; Codding et al., 2020). Possible reasons for this inconsistency include the comparably lower number of AMPPS sessions that the students received in this study and the specific AMPPS units that they received and how these units corresponded with the selected measures.

Word-problem-solving performance showed more promising results according to visual analyses, especially for Students 1, 2, 3, and 4. Existing research published about AMPPS has not specifically investigated improvements in word-problem-solving performance following an intervention, but prior research suggests that the word-problem instructional strategies used within AMPPS—including schema-based instruction focused on problem types, attack strategies, and diagrams—are associated with improved word-problem-solving outcomes (Fuchs et al., 2010; Jitendra et al., 2007; Jitendra & Hoff, 1996; Jitendra & Xin, 1997; Myers et al., 2025; Powell et al., 2020, 2023).

When examining performance across all students using NAP effect size analyses, statistically significant improvements were found for both computation and word-problem solving (see bottom row of Table 2). Although these analyses are supplemental to the primary visual analyses, the NAP data are important to consider because they suggest that, across the small sample of five students, significant improvements were identified with appropriate and commonly used effect size analyses for single-case designs. This finding is consistent with the only other evaluation of AMPPS-1:1 (Newson et al., 2025). This previous study similarly showed that, across all students, scores on basic math computation fluency were better at a statistically significant level during intervention compared to baseline conditions. Yet the NAP findings in the present study must also be interpreted cautiously because, when using NAP analyses to examine individual student data on the A-PM and CBM-WPS measures, only a few students had effect sizes that were statistically significant or approached statistical significance (a finding that was not consistent with those of Newson et al., where all but one student had statistically significant improvements).

As shown in Table 4, all participants demonstrated greater middle- to end-of-year benchmark improvements than their grade-level peers nationwide. Additionally, all but one student improved more than their same-school peers when compared to both all students and peers with math difficulties. The magnitude of improvement was also quite notable for several students. For example, Students 2 and 5 had EOY scores on the A-Comp measure that were 21 and 27 points higher than what would be expected of their respective end-of-year scores based on national norms. As previously stated, these comparisons were exploratory in nature and intended to provide additional and meaningful descriptive data that are often available and useful to practitioners. Given the small sample size and our study design, these findings should not be interpreted as causal effects. Alternative explanations for our participants’ stronger relative growth on the A-Comp measure (e.g., maturation, concurrent instruction) should be examined in follow-up studies.

It is noteworthy that we found that all students exceeded expected growth compared to national norms, particularly from the lens of a practitioner. Although weekly progress monitoring is a common component of intervention delivery and evaluation, school practitioners (e.g., classroom teachers, intervention specialists, school psychologists) often look at individual students’ performance on benchmark assessments relative to national and school-based norms. This assessment of benchmark data often serves as a meaningful gauge of students’ performance and responsiveness to interventions and usually offers a more valid assessment compared to trends in weekly progress monitoring (Begeny et al., 2023a; Crawford, 2014; Shapiro & Clemens, 2009).

Finally, students’ math self-concept did not appear to be influenced by receiving AMPPS. This finding may initially appear inconsistent with prior research showing that interventions can sometime improve students’ self-beliefs regarding their math skills (Place et al., 2023). However, students in this study did not make clear and consistent improvements on the progress monitoring probes, particularly those focused on computation, so one might contend that the students were not yet observing or experiencing regular improvements in their weekly math performance, and this may have influenced possible improvements in math self-concept. The lack of change in self-concept could have also been influenced by the relatively lower number of AMPPS sessions that students ultimately received during the study—as compared to past studies evaluating AMPPS. In this way, the reports about self-concept align with data from students’ weekly progress monitoring probes and their exposure to the intervention. Additionally, the prior literature suggests that self-concept is more resistant to change than self-efficacy (Bong & Skaalvik, 2003), which likewise could have impacted potential improvements in this area. Collectively, several factors help to explain why student improvements in math self-concept were not found in this study.

### 4.1. Considerations, Limitations, and Future Research Directions

In this section, we highlight the primary study considerations, limitations, and general suggestions for future research. We then describe how future AMPPS-1:1 research would benefit from one or more systematic replication studies—given the unique characteristics and benefits of replication studies.

#### 4.1.1. Intervention Dosage

As described previously, students in the present study received 15–21 total AMPPS intervention sessions over the course of the study. This session count (i.e., dosage) reflects a meaningful number of intervention sessions to evaluate potential treatment effects (Begeny, 2011; DeFouw et al., 2019)—and, as noted previously, the methodology of our study meets nearly every recommendation for a highly rigorous SCD, as described by a team of SCD experts and reported through the United States IES (see What Works Clearinghouse, 2022). However, AMPPS dosage of 15–21 sessions is notably lower than the ~30–50 sessions recommended by the developers of AMPPS and lower than the number of sessions that students received in prior AMPPS studies. For instance, students in the evaluation of AMPPS-1:1 by Newson et al. (2025) each received 26–30 sessions. Similarly, students in the evaluation of AMPPS-SG by Begeny et al. (2020) received approximately 40 sessions, and students in the study by Codding et al. (2020) received an average of 28.4 sessions. Consistent with the overall low number of sessions, this also meant that students generally received less than the recommended cadence/frequency of AMPPS implementation per week, which is recommended to be at least three times per week until students receive 30–50 total sessions. In the present study, due to the factors described earlier, students often only received two sessions per week. Had they predominantly received three sessions per week and, on occasion, only two sessions per week, this would have still been consistent with current recommendations for AMPPS as long as a total of 30–50 sessions was achieved. However, students in our study often only received two sessions per week. Although this weekly cadence is important to experimentally evaluate (as we did in the present study), it is not consistent with the “gold standard” recommendations for AMPPS-specific dosage or weekly frequency. Overall, our study provides a meaningful evaluation of AMPPS with the dosage and cadence that students received, but, as discussed later in the Implications for Practice section, there are important dosage implications to consider for research and practice.

MTSS/RtI is rooted in the idea that students who demonstrate more needs require more intensive intervention and with proper frequency and continuity (e.g., Barnett et al., 2004; Begeny, 2011; Begeny et al., 2023a; Ledford et al., 2018). Yet a systematic evaluation of specific components of intervention intensity—which includes factors such as the treatment session length (minutes), treatment session frequency (per day/week), and total treatment duration (weeks)—is not always readily available (Codding & Lane, 2015; DeFouw et al., 2019). Future research and practice (across all school-based interventions) should seek to determine the number of sessions and/or number of weeks of intervention at which students begin to see meaningful changes to performance, as such information will not only shed light on optimal intervention delivery but also provide critical information to educators regarding the practical utility of an intervention given their students’ needs and available resources within the school (Richardson, 2018). These types of determinations and considerations are available for some interventions (see Begeny, 2011; Begeny et al., 2023a), but this type of work is still needed for AMPPS and several other school-based interventions.

#### 4.1.2. Measurement of Outcome Variables

The results of the present study may have also been influenced by issues related to the alignment between measurement and targeted skills during intervention. As a function of students’ specific needs, each participant started in Unit D of the AMPPS Curriculum. Given the limited number of sessions that each student received, no student reached Unit H (i.e., complex computation with regrouping, as shown in Appendix A). As a result, A-PM may not have been the best measure for progress monitoring, as it includes several items that were not the specific target of intervention (based on the AMPPS units that the students received). From an experimental procedure and design perspective, assessment should not be too narrowly focused or overly aligned with the intervention (What Works Clearinghouse, 2022), but, in this case, students perhaps did not receive enough AMPPS sessions across enough units to demonstrate more meaningful improvements on the A-PM, which limits the extent to which the null functional relation can be interpreted as evidence of intervention ineffectiveness.

Additionally, the present study utilized a shortened version of the CBM-WPS probe compared to what was validated in previous research. As previously stated, the probes were shortened to accommodate time restraints related to the participating school’s daily schedule. Furthermore, to have a sufficient number of probes to monitor progress at the desired frequency throughout the baseline and intervention phases, additional probes were created that mirrored the structure and format of the original probes. As such, the specific probes used in the present study have not been researched, although, anecdotally, they had good face validity and were feasible to implement. Nevertheless, the reliability and validity of the shortened probes have not been established, and measurement limitations may have contributed to the observed variability in some students’ performance. Given the limited options for progress monitoring tools focused on word-problem solving, additional work is needed to develop and evaluate tools with strong psychometrics that practitioners and researchers can use when evaluating math interventions that can impact word-problem solving.

Finally, self-concept was selected as the variable of interest in the present study given the closer alignment of this construct with the National Research Council’s (2001) definition of productive disposition. However, research surrounding self-beliefs is complicated by inconsistently defined terminology used in math scholarship (e.g., Bong & Skaalvik, 2003; Lee, 2009; Marsh et al., 2019; Zimmerman, 2000). Future research may wish to consider which of the various constructs related to self-beliefs (e.g., self-efficacy, self-concept, self-esteem, confidence) are most closely related to math performance, which constructs are most likely to be impacted by effective math intervention, and which measures most accurately capture these constructs. With AMPPS more specifically, it is recommended that future research examine whether changes in math self-beliefs are associated with a specific frequency (i.e., sessions per week) or duration (i.e., number of total weeks) of intervention, particularly given that AMPPS incorporates evidence-based motivational strategies, which have been associated with improved math performance (e.g., Begeny et al., 2020; Codding et al., 2020). The findings from such research could also provide additional insight regarding optimal intervention intensity and dosage.

#### 4.1.3. Interaction of Aforementioned Limitations with Selected Research Design

An important consideration when evaluating academic interventions is the research design. When designing a study, researchers are forced to identify the methodology that will best answer their proposed research questions, which necessitates navigating the associated pros, cons, and trade-offs of various designs. A review of the literature suggests that both SCDs and group designs have routinely been used to evaluate academic interventions (Bliss et al., 2008; Methe et al., 2012; Pellegrini et al., 2021; Riley-Tillman et al., 2020; Williams et al., 2022). More specifically, SCDs have previously been used to evaluate AMPPS, and the results have suggested positive effects (Begeny et al., 2020; Codding et al., 2020; Newson et al., 2025). SCDs allow for considering individual-specific variables, can be executed with a smaller number of participants, and do not necessitate a control group, which can be an important ethical consideration (i.e., researchers do not need to intentionally deny intervention for students who demonstrate need).

However, an important consideration for future research may be the intersection of all methodological considerations and the study design. For example, considerations for the total number of intervention sessions, as well as using measures closely aligned with the skills taught, may be especially important for SCD research, given the nature of the design and prior research indicating that immediate level changes are not typically expected for academic interventions (Barnett et al., 2004; Begeny et al., 2020).

#### 4.1.4. Systematic Replication Studies and Implications for Future Research

Given the existing research with AMPPS and key considerations of the present study, we strongly encourage systematic replication studies (SRSs) to examine similar research questions to those of the present study. SRSs are essential for understanding the generality of a given phenomenon (e.g., an effect of a school-based intervention) across a wide range of situations and conditions (Hoffmann et al., 2025; Valentine et al., 2011; V. C. Wong & Steiner, 2018), and they are commonly used in the behavioral and educational sciences to extend existing research and knowledge of interventions (e.g., Barton et al., 2020; Berg-Mortensen et al., 2022; Ruiz-García & Valero-Aguayo, 2023; K. K. Wong & Fienup, 2022). To name a few examples, SRSs help those who use, potentially benefit from, and/or evaluate an intervention to understand how intervention effects may differ depending on variations in (a) the intervention dosage; (b) the tools or processes for measuring key outcome variables; (c) the implementation setting or context (e.g., during or outside a school day, during extended school breaks); or (d) the characteristics of the interventionists or intended beneficiaries of the intervention.

Our heterogenous findings—compared to previous AMPPS studies and in light of dosage and measurement considerations—highlight a logical next step for future research: conducting an SRS of AMPPS and its effects on complex computation, word-problem solving, and constructs related to math self-efficacy or self-concept. More specifically, we hypothesize that the positive impacts of AMPPS, as shown in the visual analyses and individualized NAP effect size analyses, would have been even clearer and more consistent with past AMPPS studies if (a) students had received 30–50 AMPPS sessions with a typical implementation cadence of at least three sessions per week (often achieved when implemented across three-fourths of a school year or more) and (b) students had received all lessons within AMPPS Units E, F, G, and H (see Appendix A)—which would have added even more support for students’ learning of complex computation. With this hypothesis, we recommend a future SRS that utilizes and adequately reports (see Hoffmann et al., 2025) the same study characteristics as the present study (e.g., overall participant demographics, experimental design, setting, recruitment strategy, measures, materials, interventionists, etc.) but incorporates the two aforementioned considerations into the SRS plan.

Such an SRS would significantly contribute to knowledge about the impacts of AMPPS, but we also highlight this as a general consideration for researchers of school-based interventions, because such SRSs are essential for fully understanding intervention effects across different conditions—and SRSs are imperative for strengthening the knowledge base about the generalizability and outcome heterogeneity of an intervention. Indeed, for AMPPS and other school-based interventions, one SRS is far from sufficient for understanding the potential effects of a respective intervention. As such, we agree with other behavioral and educational researchers (e.g., Begeny, 2011; Bryan et al., 2021; Cook et al., 2016; Coyne et al., 2016; Maxwell et al., 2015; Nicolosi & Dillenburger, 2022) that multiple SRSs—including those using different types of experimental designs—should be intentionally and effectively conducted to strengthen the research and knowledge base about a given intervention. Only with such research can practitioners be adequately informed about key issues related to intervention generalizability, implementation requirements, and potential for sustainability.

Furthermore, because many countries have a high percentage of students who do not have appropriate math proficiency (OECD, 2024), this likewise opens opportunities for SRSs. In short, because we cannot make assumptions about the effects of math interventions being larger or smaller across different national and geographic contexts (Begeny, 2018; Thalmayer et al., 2021), another area for future research and SRSs would be to examine the effects of AMPPS (or similar math interventions) in geographical contexts outside the United States. Similarly, best practices for program adaptation and/or translation (e.g., Martins et al., 2023) could potentially be used with AMPPS to examine program effects when used in different languages.

### 4.2. Implications for Practice

Although the findings of the present study do not unequivocally support the effectiveness of AMPPS-1:1 under the circumstances with which AMPPS was implemented (e.g., lower than recommended sessions) and how progress was measured (e.g., limitations of the A-PM and CBM-WPS probes), there are still some important implications for education practitioners (e.g., classroom teachers, intervention specialists, school psychologists) to consider in light of the data obtained. As illustrated in the present study, even programs that are evidence-based and have shown positive effects in prior research may not show positive impacts at all times. In fact, heterogeneous findings are common in intervention research (Conaway et al., 2024; Richardson, 2018; Williams et al., 2022), and other researchers have argued that heterogeneity should become an even greater focus of research (Bryan et al., 2021; Coyne et al., 2016; Shrout & Rodgers, 2018).

In other words, an intervention program may be effective, but only under certain conditions—such as those related to frequency, modality, dosage, and setting—and intervention researchers and developers need to help program users (e.g., teachers and intervention specialists) to understand the contexts under which interventions are most likely to be effective (e.g., Begeny et al., 2023a; Foster et al., 2023; Hoffmann et al., 2025). In this way, our results underscore the importance of sufficient intervention dosage, and the mixed pattern of effects may reflect the lower-than-recommended total session count and sessions per week rather than program ineffectiveness.

Furthermore, the reported effectiveness of an intervention may vary based on the type of assessment used (de Boer et al., 2014; Slavin & Madden, 2011), such as progress monitoring measures versus benchmark assessments in school-based academic intervention research. Finally, one should not presume that every student receiving an intervention—even an intervention with many studies showing its efficacy—would respond in the same way (Deno & Fuchs, 1987; Cook et al., 2016). Such nuances are critical for researchers to keep in mind as they evaluate intervention programs, as their findings are often considered by educators seeking practical guidance (Coyne et al., 2016; Nicolosi & Dillenburger, 2022; Penuel et al., 2017; Richardson, 2018).

To best understand the impacts of a program in their specific context, educators should consider a variety of dimensions beyond individuals’ performance, including intervention frequency, implementation fidelity, and individual differences (Weiss et al., 2014). Furthermore, such factors should be continuously monitored over time (Gersten et al., 2009a; McKenna & Parenti, 2017), and multiple methods should be used to make these evaluations (Joyce & Cartwright, 2020; Shapiro & Clemens, 2009; Valentine et al., 2011).

## 5. Conclusions

Despite idiosyncratic findings and inconsistencies with prior AMPPS studies, this study extends the literature on the AMPPS program and contributes to growing discussions regarding the heterogeneity and generalizability of intervention research within the psychological and educational sciences. Many researchers have emphasized the importance of understanding and publishing heterogenous, mixed, and/or nonsignificant findings (e.g., Bryan et al., 2021; Hoffmann et al., 2025; Maxwell et al., 2015), and, as Shrout and Rodgers (2018) state, there is a strong need for “more complete disclosure of nonsignificant as well as statistically significant findings” (p. 487). Future research, such as SRSs, is needed to identify and clearly define the factors that influence the impacts of receiving AMPPS on students’ math performance, including those related to intervention intensity, as well as student- and school-level characteristics. This same need is arguably true for all school-based interventions that educators consider when trying to promote students’ social–emotional and academic success.

## Figures and Tables

**Table 1:** Means and standard deviations for each student by phase.

Participant	Computation (A-PM)		Word-Problem Solving (CBM-WPS)	
	Baseline *M* (*SD*)	Intervention *M* (*SD*)	Baseline *M* (*SD*)	Intervention *M* (*SD*)
Student 1	5.7 (0.3)	6.4 (1.7)	0.3 (0.6)	3.3 (2.2)
Student 2	11.8 (1.6)	12.7 (1.9)	4.7 (2.5)	5.8 (1.9)
Student 3	4.0 (0.4)	6.6 (2.8)	2.0 (1.6)	4.5 (1.9)
Student 4	4.7 (1.9)	5.6 (1.5)	0.4 (0.9)	2.2 (1.5)
Student 5	17.6 (0.7)	16.3 (2.2)	7.2 (1.3)	7.6 (1.9)

**Table 2:** NAP effect size analyses for single-case designs.

Participant	Computation (A-PM)		Word-Problem Solving (CBM-WPS)	
	NAP	*p*-Value	NAP	*p*-Value
Student 1	0.63	0.499	0.93 ^M^	0.034 *
Student 2	0.68	0.353	0.59	0.644
Student 3	0.75	0.174	0.85	0.070 **
Student 4	0.76	0.144	0.85	0.055 **
Student 5	0.32	0.273	0.61	0.478
Across all students	0.61 ^S^	<0.001 *	0.76 ^M^	<0.001 *

NAP = nonoverlap of all pairs effect size analyses. For statistically significant NAP, S = small effect size, M = medium effect size. * Statistically significant with *p* < 0.05. ** Approaching statistical significance.

**Table 3:** School-based norms on the A-Comp, by grade, based on the study participants’ school.

Grade	*n*	Average MOY Score	Average EOY Score	Average Change
3	51	23.4	28.8	5.4
4	19 *	34.6	48.4	13.8
5	43	37.8	57.6	19.7

Note. MOY = middle of year; EOY = end of year. All data were collected in the same year that the study took place. * Missing one 4th-grade classroom due to a data collection error at MOY.

**Table 4:** Student performance on the A-Comp compared to school-based and national norms.

Participant	Grade	MOY Score (Percentile)	EOY Score (Percentile)	Change	Expected EOY *
Student 1	3	11 (10)	22 (26)	11 ^a,b,c^	15
Student 2	4	13 (8)	42 (37)	29 ^a,b,c^	21
Student 3	3	11 (10)	21 (23)	10 ^a,b,c^	15
Student 4	3	5 (2)	9 (3)	4 ^a^	7
Student 5	5	30 (19)	61 (48)	31 ^a,b,c^	34

Note. MOY = middle of year; EOY = end of year. * Acadience national norms (Gray et al., 2019a) provide scores and associated percentile ranks at BOY, MOY, and EOY for the A-Comp. Expected EOY was determined by identifying the percentile rank associated with each student’s MOY score and then identifying the EOY score associated with the same percentile rank. For example, Student 1 scored 11 at MOY, which falls at the 10th percentile. According to the national norms supplied by Acadience, a score of 15 at EOY falls at the 10th percentile, making 15 the “expected EOY” score for Student 1. ^a^ Greater than national norm. ^b^ Greater than school-based norm. ^c^ Greater than school-based norm for students who performed well below benchmark on the A-Comp at MOY.

**Table 5:** Mean scores and standard deviations for each item of the SDQI.

Item	Pre-Test *M* (*SD*)	Post-Test *M* (*SD*)
1. I get good grades in math	3.2 (1.3)	3.2 (1.1)
2. I learn things quickly in math	3.2 (1.3)	3.4 (1.3)
3. I am good at math	3.2 (0.8)	3.2 (0.4)
4. Work in math is easy for me	2.8 (1.1)	2.6 (1.1)
Average score	3.1 (0.7)	3.1 (0.9)

Note. Items were scored on a scale from 1 to 5, with higher numbers reflecting more positive attitudes and lower numbers reflecting more negative attitudes.

## Data Availability

The data presented in this study are available on request from the corresponding author.

## Abbreviations

The following abbreviations are used in this manuscript:

A-Comp	Acadience Grade-Level Computation Benchmark Assessment
A-PM	Acadience Level 2 Computation Progress Monitoring Probes
AMPPS	Accelerating Mathematics Performance with Practice Strategies
CBM-WPS	Curriculum-Based Measure of Word Problem Solving
DCPM	Digits Correct Per Minute
EOY	End of Year
MOY	Middle of Year
NAP	Nonoverlap of All Pairs
SRS	Systematic Replication Study

## Appendix A

*List of the Eight Instructional Units Included within the AMPPS Programs*

Unit A: Addition with Sums 0 to 10

Unit B: Subtraction with Minuends 0 to 10

Unit C: Addition and Subtraction Fact Families 0 to 10

Unit D: Addition and Subtraction 11 to 18

Unit E: Complex Addition of Three One-Digit Numbers

Unit F: Complex Addition and Subtraction 2 × 1 Digit

Unit G: Complex Addition and Subtraction without Regrouping

Unit H: Complex Addition and Subtraction with Regrouping

Note. The above units are used for both the AMPPS One-on-One (AMPPS-1:1) Program and the AMPPS Program for Small Instructional Groups (AMPPS-SG).

## Appendix B

*AMPPS-1:1 Implementation Checklist Example: Unit G*

## References

[B1-behavsci-16-00432] Arens A. K., Marsh H. W., Pekrun R., Lichtenfeld S., Murayama K., vom Hofe R. (2017). Math self-concept, grades, and achievement test scores: Long-term reciprocal effects across five waves and three achievement tracks. Journal of Educational Psychology.

[B2-behavsci-16-00432] Arens A. K., Yeung A. S., Craven R. G., Hasselhorn M. (2011). The twofold multidimensionality of academic self-concept: Domain specificity and separation between competence and affect components. Journal of Educational Psychology.

[B3-behavsci-16-00432] Barnett D. W., Daly E. J., Jones K. M., Lentz F. E. (2004). Response to intervention: Empirically based special service decisions from single-case designs of increasing and decreasing intensity. The Journal of Special Education.

[B4-behavsci-16-00432] Barton E. E., Velez M., Pokorski E. A., Domingo M. (2020). The effects of email performance-based feedback delivered to teaching teams: A systematic replication. Journal of Early Intervention.

[B5-behavsci-16-00432] Begeny J. C. (2011). Effects of the helping early literacy with practice strategies (HELPS) reading fluency program when implemented at different frequencies. School Psychology Review.

[B6-behavsci-16-00432] Begeny J. C. (2018). A working definition and conceptual model of internationalization for school and educational psychology. Psychology in the Schools.

[B7-behavsci-16-00432] Begeny J. C., Codding R. S., Wang J., Hida R. M., Patterson S. L., Kessler S., Fields-Turner F., Ramos K. A. (2020). An analysis of motivation strategies used within the small-group accelerating mathematics performance through practice strategies (AMPPS-SG) program. Psychology in the Schools.

[B8-behavsci-16-00432] Begeny J. C., Laugle K. M., Krouse H. E., Lynn A. E., Tayrose M. P., Stage S. A. (2010). A control-group comparison of two reading fluency programs: The helping early literacy with practice strategies (HELPS) program and the great leaps K–2 reading program. School Psychology Review.

[B9-behavsci-16-00432] Begeny J. C., Mitchell R. C., Whitehouse M. H., Harris C. F., Stage S. A. (2011). Effects of the HELPS reading fluency program when implemented by classroom teachers with low-performing second grade students. Learning Disabilities Research and Practice.

[B10-behavsci-16-00432] Begeny J. C., Newson N. K., Davidson F. L., O’Neal C. S., Durling J. M., Coolong-Chaffin M., Hawkins R. O., Axelrod M. I. (2023a). Helping early literacy with practice strategies (HELPS) program. Reading intervention case studies for school psychologists.

[B11-behavsci-16-00432] Begeny J. C., Wang J., Levy R. A., Sanetti L. M., Loehman J., Rodriguez K. (2023b). Considering the implementation research-to-practice gap: An experimental evaluation of intervention-general methods for assessing and supporting intervention fidelity through coaching. Journal of School Psychology.

[B12-behavsci-16-00432] Berg-Mortensen C., Tangen L., Strømgren B. (2022). Examining the relationship between component and composite frequency and natural gain in basic multiplication: A systematic replication of Lin and Kubina. Journal of Behavioral Education.

[B13-behavsci-16-00432] Bice-Urbach B. J., Kratochwill T. R. (2016). Teleconsultation: The use of technology to improve evidence-based practices in rural communities. Journal of School Psychology.

[B14-behavsci-16-00432] Bliss S. L., Skinner C. H., Hautau B., Carroll E. E. (2008). Articles published in four school psychology journals from 2000 to 2005: An analysis of experimental/intervention research. Psychology in the Schools.

[B15-behavsci-16-00432] Bong M., Skaalvik E. M. (2003). Academic self-concept and self-efficacy: How different are they really?. Educational Psychology Review.

[B16-behavsci-16-00432] Bryan C. J., Tipton E., Yeager D. S. (2021). Behavioural science is unlikely to change the world without a heterogeneity revolution. Nature Human Behaviour.

[B17-behavsci-16-00432] Byrne B. M., Bracken B. A. (1996). Academic self-concept: Its structure, measurement, and relation to academic achievement. Handbook of self-concept: Developmental, social, and clinical considerations.

[B18-behavsci-16-00432] Carpenter T. P., Fennema E., Franke M. L. (1996). Cognitively guided instruction: A knowledge base for reform in primary mathematics instruction. The Elementary School Journal.

[B19-behavsci-16-00432] Codding R. S., Begeny J. C. (2018a). The accelerating mathematics performance with practice strategies program for one-on-one instruction (AMPPS-1:1): Curriculum materials for units covering simple and complex addition and subtraction.

[B20-behavsci-16-00432] Codding R. S., Begeny J. C. (2018b). The accelerating mathematics performance with practice strategies program for small instructional groups (AMPPS-SG): Curriculum materials for units covering simple and complex addition and subtraction.

[B21-behavsci-16-00432] Codding R. S., Begeny J. C., Kromminga K. R., Edmunds R. R., Klaft J., Diggs C., Hanson-Burke A. (2020). Do motivational strategies improve the effects of a small-group math intervention program?. Journal of Applied School Psychology.

[B22-behavsci-16-00432] Codding R. S., Kromminga K. R., Newson N. K., Begeny J. C., Goodridge A. E., Ruedy A. P. (2022). The accelerating mathematics performance with practice strategies program for one-on-one instruction (AMPPS-1:1): Curriculum materials for units A through H.

[B23-behavsci-16-00432] Codding R. S., Lane K. L. (2015). A spotlight on treatment intensity: An important and often overlooked component of intervention inquiry. Journal of Behavioral Education.

[B24-behavsci-16-00432] Codding R. S., Volpe R. J., Poncy B. C. (2017). Effective math interventions: A guide to improving whole-number knowledge.

[B25-behavsci-16-00432] Conaway C., Tipton E., Artiles A. J. (2024). The value of variation: Embracing heterogeneity in intervention research. Teachers College Record.

[B26-behavsci-16-00432] Cook B. G., Collins L. W., Cook S. C., Cook L. (2016). A replication by any other name: A systematic review of replicative intervention studies. Remedial and Special Education.

[B27-behavsci-16-00432] Coyne M. D., Cook B. G., Therrien W. J. (2016). Recommendations for replication research in special education: A framework of systematic, conceptual replications. Remedial and Special Education.

[B28-behavsci-16-00432] Crawford L. (2014). The role of assessment in a response to intervention model. Preventing School Failure.

[B29-behavsci-16-00432] de Boer H., Donker A. S., van der Werf M. P. (2014). Effects of the attributes of educational interventions on students’ academic performance: A meta-analysis. Review of Educational Research.

[B30-behavsci-16-00432] DeFouw E. R., Codding R. S., Collier-Meek M. A., Gould K. M. (2019). Examining dimensions of treatment intensity and treatment fidelity in mathematics intervention research for students at risk. Remedial and Special Education.

[B31-behavsci-16-00432] Deno S. L., Fuchs L. S. (1987). Developing curriculum-based measurement systems for data-based special education problem solving. Focus on Exceptional Children.

[B32-behavsci-16-00432] Doabler C. T., Clarke B., Kosty D., Kurtz-Nelson E., Fien H., Smolkowski K., Baker S. K. (2019). Examining the impact of group size on the treatment intensity of a tier 2 mathematics intervention within a systematic framework of replication. Journal of Learning Disabilities.

[B33-behavsci-16-00432] Duncan G. J., Dowsett C. J., Claessens A., Magnuson K., Huston A. C., Klebanov P., Pagani L. S., Feinstein L., Engel M., Brooks-Gunn J., Sexton H., Duckworth K., Japel C. (2007). School readiness and later achievement. Developmental Psychology.

[B34-behavsci-16-00432] Foster J. A., Mossing K. W., Stepanek M., Wang J., Coolong-Chaffin M., Hawkins R. O., Axelrod M. I. (2023). Rewards. Reading intervention case studies for school psychologists.

[B35-behavsci-16-00432] Fuchs L. S., Newman-Gonchar R., Schumacher R., Dougherty B., Bucka N., Karp K. S., Woodward J., Clarke B., Jordan N. C., Gersten R., Jayanthi M., Keating B., Morgan S. (2021). Assisting students struggling with mathematics: Intervention in the elementary grades *(WWC 2021006)*.

[B36-behavsci-16-00432] Fuchs L. S., Powell S. R., Cirino P. T., Schumacher R. F., Marrin S., Hamlett C. L., Fuchs D., Compton D. L., Changas P. C. (2014). Does calculation or word-problem instruction provide a stronger route to prealgebraic knowledge?. Journal of Educational Psychology.

[B37-behavsci-16-00432] Fuchs L. S., Zumeta R. O., Schumacher R. F., Powell S. R., Seethaler P. M., Hamlett C. L., Fuchs D. (2010). The effects of schema-broadening instruction on second graders’ word-problem performance and their ability to represent word problems with algebraic equations: A randomized control study. The Elementary School Journal.

[B38-behavsci-16-00432] Ganley C. M., Lubienski S. T. (2016). Mathematics confidence, interest, and performance: Examining gender patterns and reciprocal relations. Learning and Individual Differences.

[B39-behavsci-16-00432] Gersten R., Beckmann S., Clarke B., Foegen A., Marsh L., Star J. R., Witzel B. (2009a). Assisting students struggling with mathematics: Response to Intervention (RtI) for elementary and middle schools *(NCEE 2009-4060)*.

[B40-behavsci-16-00432] Gersten R., Chard D. J., Jayanthi M., Baker S. K., Morphy P., Flojo J. (2009b). Mathematics instruction for students with learning disabilities: A meta-analysis of instructional components. Review of Educational Research.

[B41-behavsci-16-00432] Gray J. S., Warnock A. N., Dewey E. N., Latimer R., Wheeler C. E. (2019a). Acadience™ math national norms 2016–2017 *(Technical Report No. 25)*.

[B42-behavsci-16-00432] Gray J. S., Warnock A. N., Dewey E. N., Latimer R., Wheeler C. E. (2019b). Acadience™ math technical adequacy brief.

[B43-behavsci-16-00432] Han F. (2019). Self-concept and achievement in math among Australian primary students: Gender and culture issues. Frontiers in Psychology.

[B44-behavsci-16-00432] Hansen N., Jordan N. C., Rodrigues J. (2017). Identifying persistent learning difficulties in fractions: A longitudinal study of student growth from third through sixth grade. Contemporary Educational Psychology.

[B45-behavsci-16-00432] Hickendorff M., Torbeyns J., Verschaffel L., Fritz A., Haase V. G., Räsänen P. (2019). Multi-digit addition, subtraction, multiplication, and division strategies. International handbook of mathematical learning difficulties.

[B46-behavsci-16-00432] Hoffmann J., Twardawski M., Höhs J. M., Gast A., Pohl S., Sengewald M.-A. (2025). The design of current replication studies: A systematic literature review on the variation of study characteristics. Advances in Methods and Practices in Psychological Science.

[B47-behavsci-16-00432] Jitendra A. K., Dupuis D. N., Zaslofsky A. F. (2014). Curriculum-based measurement and standards-based mathematics: Monitoring the arithmetic word problem-solving performance of third-grade students at risk for mathematics difficulties. Learning Disability Quarterly.

[B48-behavsci-16-00432] Jitendra A. K., Griffin C. C., Deatline-Buchman A., Sczesniak E. (2007). Mathematical word problem solving in third-grade classrooms. The Journal of Educational Research.

[B49-behavsci-16-00432] Jitendra A. K., Hoff K. (1996). The effects of schema-based instruction on the mathematical word-problem-solving performance of students with learning disabilities. Journal of Learning Disabilities.

[B50-behavsci-16-00432] Jitendra A. K., Sczesniak E., Deatline-Buchman A. (2005). An exploratory validation of curriculum-based mathematical word problem-solving tasks as indicators of mathematics proficiency for third graders. School Psychology Review.

[B51-behavsci-16-00432] Jitendra A. K., Xin Y. P. (1997). Mathematical word-problem-solving instruction for students with mild disabilities and students at risk for math failure: A research synthesis. The Journal of Special Education.

[B52-behavsci-16-00432] Jordan N. C., Kaplan D., Ramineni C., Locuniak M. N. (2009). Early math matters: Kindergarten number competence and later mathematics outcomes. Developmental Psychology.

[B53-behavsci-16-00432] Joyce K. E., Cartwright N. (2020). Bridging the gap between research and practice: Predicting what will work locally. American Educational Research Journal.

[B54-behavsci-16-00432] Kratochwill T. R., Hitchcock J. H., Horner R. H., Levin J. R., Odom S., Rindskopf D. M., Shadish W. R. (2013). Single-case intervention research design standards. Remedial and Special Education.

[B55-behavsci-16-00432] Kratochwill T. R., Levin J. R. (2010). Enhancing the scientific credibility of single-case intervention research: Randomization to the rescue. Psychological Methods.

[B56-behavsci-16-00432] Ledford J. R., Lane J. D., Severini K. E. (2018). Systematic use of visual analysis for assessing outcomes in single case design studies. Brain Impairment.

[B57-behavsci-16-00432] Lee J. (2009). Universals and specifics of math self-concept, math self-efficacy, and math anxiety across 41 PISA 2003 participating countries. Learning and Individual Differences.

[B58-behavsci-16-00432] Leh J. M., Jitendra A. K., Caskie G. I., Griffin C. C. (2007). An evaluation of curriculum-based measurement of mathematics word problem solving measures for monitoring third-grade students’ mathematics competence. Assessment for Effective Intervention.

[B59-behavsci-16-00432] Levin J. R., Ferron J. M. (2021). Different randomized multiple-baseline models for different situations: A practical guide for single-case intervention researchers. Journal of School Psychology.

[B60-behavsci-16-00432] Ma X., Kishor N. (1997). Assessing the relationship between attitude toward mathematics and achievement in mathematics: A meta-analysis. Journal for Research in Mathematics Education.

[B61-behavsci-16-00432] Marsh H. W. (1986). Negative item bias in rating scales for preadolescent children: A cognitive-developmental phenomenon. Developmental Psychology.

[B62-behavsci-16-00432] Marsh H. W. (1990). Self Description Questionnaire-I (SDQI) *[Database record]*.

[B63-behavsci-16-00432] Marsh H. W., Craven R. G., Debus R. (1999). Separation of competency and affect components of multiple dimensions of academic self-concept: A developmental perspective. Merrill-Palmer Quarterly.

[B64-behavsci-16-00432] Marsh H. W., Pekrun R., Parker P. D., Murayama K., Guo J., Dicke T., Arens A. K. (2019). The murky distinction between self-concept and self-efficacy: Beware of lurking jingle-jangle fallacies. Journal of Educational Psychology.

[B65-behavsci-16-00432] Martins M. A., Begeny J. C., Aparecida Capellini S. (2023). Translation and cultural adaptation of the HELPS reading fluency program into Brazilian Portuguese. A report of systematic adaptation processes and initial evidence of efficacy. Frontiers in Psychology.

[B66-behavsci-16-00432] Maxwell S. E., Lau M. Y., Howard G. S. (2015). Is psychology suffering from a replication crisis? What does “failure to replicate” really mean?. American Psychologist.

[B67-behavsci-16-00432] McKenna J. W., Parenti M. (2017). Fidelity assessment to improve teacher instruction and school decision making. Journal of Applied School Psychology.

[B68-behavsci-16-00432] Methe S. A., Kilgus S. P., Neiman C., Riley-Tillman T. C. (2012). Meta-analysis of interventions for basic mathematics computation in single-case research. Journal of Behavioral Education.

[B69-behavsci-16-00432] Miller A. H., Espinas D. R., McNeish D., Barnes M. A. (2025). Dosage response in intensive mathematics interventions for early elementary students with or at-risk for mathematics learning disability. Educational Psychology Review.

[B70-behavsci-16-00432] Myers J. A., Arsenault T. L., Powell S. R., Witzel B. S., Tanner E., Pigott T. D. (2025). Considerations for intensifying word-problem interventions for students with MD: A qualitative umbrella review of relevant meta-analyses. Journal of Learning Disabilities.

[B71-behavsci-16-00432] Namkung J. M., Fuchs L. S., Koziol N. (2018). Does initial learning about the meaning of fractions present similar challenges for students with and without adequate whole-number skill?. Learning and Individual Differences.

[B72-behavsci-16-00432] National Center for Education Statistics (n.d.). The nation’s report card: NAEP data explorer.

[B73-behavsci-16-00432] National Center for Education Statistics (2024). The nation’s report card: Mathematics 2024.

[B74-behavsci-16-00432] National Governors Association Center for Best Practices, Council of Chief State School Officers (2010). Common sore state standards for mathematics.

[B75-behavsci-16-00432] National Mathematics Advisory Panel (2008). Foundations for success: The final report of the National Mathematics Advisory Panel.

[B76-behavsci-16-00432] National Research Council (2001). Adding it up: Helping children learn mathematics.

[B77-behavsci-16-00432] Nelson G., Powell S. R. (2018). A systematic review of longitudinal studies of mathematics difficulty. Journal of Learning Disabilities.

[B78-behavsci-16-00432] Nelson P. M., Parker D. C., Zaslofsky A. F. (2016). The relative value of growth in math fact skills across late elementary and middle school. Assessment for Effective Intervention.

[B79-behavsci-16-00432] Newson N. K., Begeny J. C., Wang J., Polanco Jordán Y., Codding R. S., Kromminga K. R. (2025). Effects of the accelerating mathematics performance with practice strategies (AMPPS) intervention program when delivered during summer in a one-on-one virtual format. Psychology in the Schools.

[B80-behavsci-16-00432] Nicolosi M., Dillenburger K. (2022). The University of California at Los Angeles–Young Autism Project: A systematic review of replication studies. Behavioral Interventions.

[B81-behavsci-16-00432] OECD (2024). Policy sub-issue from the organisation for economic co-operation and development: Mathematics literacy.

[B82-behavsci-16-00432] Parker R. I., Vannest K. (2009). An improved effect size for single-case research: Nonoverlap of all pairs. Behavior Therapy.

[B83-behavsci-16-00432] Parsons S., Bynner J. (1997). Numeracy and employment. Education + Training.

[B84-behavsci-16-00432] Pellegrini M., Lake C., Neitzel A., Slavin R. E. (2021). Effective programs in elementary mathematics: A meta-analysis. AERA Open.

[B85-behavsci-16-00432] Penuel W. R., Briggs D. C., Davidson K. L., Herlihy C., Sherer D., Hill H. C., Farrell C., Allen A.-R. (2017). How school and district leaders access, perceive, and use research. AERA Open.

[B86-behavsci-16-00432] Philipp R. A., Siegfried J. M. (2015). Studying productive disposition: The early development of a construct. Journal of Mathematics Teacher Education.

[B87-behavsci-16-00432] Place K., Gothro A., Chojnacki G., Conroy K. (2023). Testing new approaches to math tutoring: Lessons from eight evaluations.

[B88-behavsci-16-00432] Powell S. R., Berry K. A., Benz S. A. (2020). Analyzing the word-problem performance and strategies of students experiencing mathematics difficulty. Journal of Mathematical Behavior.

[B89-behavsci-16-00432] Powell S. R., Bouck E. C., Sutherland M., Clarke B., Arsenault T. L., Freeman-Green S. (2023). Essential components of math instruction. Teaching Exceptional Children.

[B90-behavsci-16-00432] Powell S. R., Fuchs L. S., Cirino P. T., Fuchs D., Compton D. L., Changas P. C. (2015). Effects of a multitier support system on calculation, word problem, and prealgebraic performance among at-risk learners. Exceptional Children.

[B91-behavsci-16-00432] Powell S. R., Fuchs L. S., Fuchs D. (2010). Embedding number-combinations practice within word-problem tutoring. Intervention in School and Clinic.

[B92-behavsci-16-00432] Resnick I., Jordan N. C., Hansen N., Rajan V., Rodrigues J., Siegler R. S., Fuchs L. S. (2016). Developmental growth trajectories in understanding of fraction magnitude from fourth through sixth grade. Developmental Psychology.

[B93-behavsci-16-00432] Richardson M. (2018). An overview of evidence-based literacy interventions in North Carolina.

[B94-behavsci-16-00432] Riley-Tillman T. C., Burns M. K., Kilgus S. P. (2020). Evaluating educational interventions: Single-case design for measuring response to intervention.

[B95-behavsci-16-00432] Rittle-Johnson B., Schneider M., Kadosh R. C., Dowker A. (2015). Developing conceptual and procedural knowledge of mathematics. Oxford handbook of numerical cognition.

[B96-behavsci-16-00432] Ruiz-García A., Valero-Aguayo L. (2023). Adults with animal phobia: Systematic replication of clinical cases. Behaviour Change.

[B97-behavsci-16-00432] Running K., Codding R. S., Varma S., Rao V. N. V., Wackerle-Hollman A. (2023). Comparing the effects of concepts-first and iterative fraction instruction sequences: A randomized controlled trial. Elementary Schol Journal.

[B98-behavsci-16-00432] Sanetti L. M. H., Collier-Meek M. A. (2014). Increasing the rigor of treatment integrity assessment: An empirical comparison of direct observation and permanent product review methods. Journal of Behavioral Education.

[B99-behavsci-16-00432] Shapiro E. S., Clemens N. H. (2009). A conceptual model for evaluating system effects of response to intervention. Assessment for Effective Intervention.

[B100-behavsci-16-00432] Shrout P. E., Rodgers J. L. (2018). Psychology, science, and knowledge construction: Broadening perspectives from the replication crisis. Annual Review of Psychology.

[B101-behavsci-16-00432] Slavin R., Madden N. A. (2011). Measures inherent to treatments in program effectiveness reviews. Journal of Research on Educational Effectiveness.

[B102-behavsci-16-00432] Thalmayer A. G., Toscanelli C., Arnett J. J. (2021). The neglected 95% revisited: Is American psychology becoming less American?. The American Psychologist.

[B103-behavsci-16-00432] Valentine J. C., Biglan A., Boruch R. F., Castro F. G., Collins L. M., Flay B. R., Kellam S., Mościcki E. K., Schinke S. P. (2011). Replication in prevention science. Prevention Science.

[B104-behavsci-16-00432] Valentine J. C., DuBois D. L., Cooper H. (2004). The relation between self-beliefs and academic achievement: A meta-analytic review. Educational Psychologist.

[B105-behavsci-16-00432] Vannest K. J., Parker R. I., Gonen O., Adiguzel T. (2016). Single case research: Web based calculators for SCR analysis *(Version 2.0) [Web-based application]*.

[B106-behavsci-16-00432] Vigna G., Ghidoni E., Burgio F., Danesin L., Angelini D., Benavides-Varela S., Semenza C. (2022). Dyscalculia in early adulthood: Implications for numerical activities of daily living. Brain Sciences.

[B107-behavsci-16-00432] Villarreal V., Castro M. J., Umaña I., Sullivan J. R. (2017). Characteristics of intervention research in school psychology journals: 2010–2014. Psychology in the Schools.

[B108-behavsci-16-00432] Weiss M. J., Bloom H. S., Brock T. (2014). A conceptual framework for studying the sources of variation in program effects. Journal of Policy Analysis and Management.

[B109-behavsci-16-00432] What Works Clearinghouse (2022). What works clearinghouse procedures and standards handbook, version 5.0.

[B110-behavsci-16-00432] Wheeler C. E., Lembke E. S., Richards-Tutor C., Wallin J., Good R. H., Dewey E. N., Warnock A. N. (2019). Acadience™ math assessment manual.

[B111-behavsci-16-00432] Williams R., Citkowicz M., Miller D. I., Lindsay J., Walters K. (2022). Heterogeneity in mathematics intervention effects: Evidence from a meta-analysis of 191 randomized experiments. Journal of Research on Educational Effectiveness.

[B112-behavsci-16-00432] Wong K. K., Fienup D. M. (2022). Units of analysis in acquisition-performance criteria for “mastery”: A systematic replication. Journal of Applied Behavior Analysis.

[B113-behavsci-16-00432] Wong V. C., Steiner P. M. (2018). Replication designs for causal inference *(EdPolicyWorks Working Paper Series, Issue 62)*.

[B114-behavsci-16-00432] Wu H., Guo Y., Yang Y., Zhao L., Guo C. (2021). A meta-analysis of the longitudinal relationship between academic self-concept and academic achievement. Educational Psychology Review.

[B115-behavsci-16-00432] Zimmerman B. J. (2000). Self-efficacy: An essential motive to learn. Contemporary Educational Psychology.

